# Dapagliflozin in patients with critical illness: rationale and design of the DEFENDER study

**DOI:** 10.5935/2965-2774.20230129-en

**Published:** 2023

**Authors:** Caio de Assis Moura Tavares, Luciano César Pontes de Azevedo, Álvaro Rea-Neto, Niklas Söderberg Campos, Cristina Prata Amendola, Ricardo Reinaldo Bergo, Amanda Christina Kozesinski-Nakatani, Paula Geraldes David-João, Glauco Adrieno Westphal, Mário Roberto Rezende Guimarães Júnior, Suzana Margareth Ajeje Lobo, Marcos Soares Tavares, Marianna Deway Andrade Dracoulakis, Guilherme Martins de Souza, Guacyra Margarita Batista de Almeida, Otavio Celso Eluf Gebara, Pablo Oscar Tomba, Camila Santos N Albuquerque, Mariana Castaldi Ramalho Silva, Adriano José Pereira, Lucas Petri Damiani, Thiago Domingos Corrêa, Ary Serpa-Neto, Otavio Berwanger, Fernando Godinho Zampieri

**Affiliations:** 1 Hospital Israelita Albert Einstein - São Paulo (SP), Brazil; 2 Centro de Estudos e de Pesquisas em Terapia Intensiva - Curitiba (PR), Brazil; 3 Hospital M´Boi Mirim - São Paulo (SP), Brazil; 4 Hospital de Câncer de Barretos - Barretos (SP), Brazil; 5 Hospital Santa Lucia - Poços de Caldas (MG), Brazil; 6 Centro Hospitalar Unimed - Joinville (SC), Brazil; 7 Santa Casa de Misericórdia de Barretos - Barretos (SP), Brazil; 8 Hospital de Base, Faculdade de Medicina de São José do Rio Preto - São José do Rio Preto (SP), Brazil; 9 Hospital Nove de Julho - São Paulo (SP), Brazil; 10 DASA Hospítal da Bahia - Salvador (BA), Brazil; 11 Hospital Vila Santa Catarina - São Paulo (SP), Brazil; 12 Hospital de Emergência Dr. Daniel Houly - Arapiraca (AL), Brazil; 13 DASA Hospital Santa Paula - São Paulo (SP), Brazil; 14 Hospital de Amor de Jales - Jales (SP), Brazil

**Keywords:** Critical illness, Sodium-glucose transporter 2 inhibitors, Organ dysfunction, Critical care outcomes

## Abstract

**Background:**

Critical illness is a major ongoing health care burden worldwide and is associated with high mortality rates. Sodium-glucose cotransporter-2 inhibitors have consistently shown benefits in cardiovascular and renal outcomes. The effects of sodium-glucose cotransporter-2 inhibitors in acute illness have not been properly investigated.

**Methods:**

DEFENDER is an investigator-initiated, multicenter, randomized, open-label trial designed to evaluate the efficacy and safety of dapagliflozin in 500 adult participants with acute organ dysfunction who are hospitalized in the intensive care unit. Eligible participants will be randomized 1:1 to receive dapagliflozin 10mg plus standard of care for up to 14 days or standard of care alone. The primary outcome is a hierarchical composite of hospital mortality, initiation of kidney replacement therapy, and intensive care unit length of stay, up to 28 days. Safety will be strictly monitored throughout the study.

**Conclusion:**

DEFENDER is the first study designed to investigate the use of a sodium-glucose cotransporter-2 inhibitor in general intensive care unit patients with acute organ dysfunction. It will provide relevant information on the use of drugs of this promising class in critically ill patients.

ClinicalTrials.gov registry NCT05558098

## INTRODUCTION

Critical illness is a major global challenge, with mortality rates after intensive care unit (ICU) admission reaching as high as 22%, according to international estimates.^(1)^ Despite this alarming issue for health care systems, no specific therapy has yet been shown to improve outcomes in unselected patients with acute organ dysfunction in the ICU.

Sodium-glucose cotransporter-2 (SGLT2) inhibitors have demonstrated a consistent reduction in cardiovascular and kidney outcomes in several randomized clinical trials (RCTs) across a range of clinical settings, including type 2 diabetes mellitus,^(2)^ heart failure with reduced and preserved ejection fraction,^(3-6)^ acute heart failure,^(7)^ and chronic kidney disease.^(8,9)^ Some of the postulated effects of this drug class^(10)^ may also positively impact multiple deleterious pathways of acute illness and protect against organ injury and failure. Plausible mechanisms that may be involved-particularly in patients with sepsis^(11)^- include improved metabolic efficiency^(12,13)^ and endothelial function,^(14)^ inhibition of pro-inflammatory pathways^(15)^ and sympathetic activity,^(16)^ decreasing production of reactive oxygen species,^(17)^ and increased erythropoietin production.^(18)^ Experimental animal models of acute injury provide data that support the existence of an overlap between the effects of SGLT2 inhibitors and protection against organ dysfunction. Sodium-glucose cotransporter-2 inhibition prevented renal injury and reduced biomarkers of systemic inflammation^(19)^ and pathological findings of lung injury^(20)^ in lipopolysaccharide-induced inflammation models.

In humans, the DARE-19 trial^(21)^ provided pivotal information to support the use of SGLT2 inhibitors in major acute illness; dapagliflozin numerically reduced the event rates of the coprimary prevention outcome (a composite of new or worsening organ dysfunction or death) in hospitalized COVID-19 patients when compared with placebo. Although the results of the trial failed to meet statistical significance for the efficacy outcome, the use of dapagliflozin was well tolerated, associated with a low rate of diabetic ketoacidosis, and did not lead to an increase in serious adverse events despite previous concerns.^(22)^

The DEFENDER study (“*Estudo Clínico RanDomizado Avaliando a Eficácia da DapagliFlozina em Pacientes IntErNaDos em Estado CRítico*”) was designed to assess the efficacy and safety of repurposing dapagliflozin for unselected critically ill patients with acute organ dysfunction.

## METHODS

DEFENDER (ClinicalTrials.gov unique identifier NCT05558098) is an investigator-initiated trial coordinated and sponsored by the Academic Research Organization (ARO) of the *Hospital Israelita Albert Einstein*, funded through the *Programa de Apoio ao Desenvolvimento Institucional do Sistema Único de Saúde* (PROADI-SUS) from the Brazilian Ministry of Health.

The study coordinating site is responsible for overseeing all trial operations (start-up activities, regulatory affairs, site management, data management, and scientific oversight). The steering committee (SC) comprises coordinating center members and academic leaders responsible for supervising the study’s progress, monitoring, and considering recommendations from the data monitoring and safety board (DSMB). SC members will also plan academic publications, draft and review the study manuscript, and present study results at scientific meetings. To ensure trial participants’ safety, the DMSB members are independent experts appointed to the committee, namely, Dr. Paul Young (chair), an intensivist and clinical researcher; Prof. Carol Hodgson, a physiotherapist specialist in intensive care and clinical trial; and Prof. Michael Bailey, a biostatistician.

The study is being conducted in accordance with Good Clinical Practice guidelines. Prior to study initiation or implementation of changes, the study protocol and all amendments will be approved by Ethical Committees at each participating site. Participants or their legal representatives will provide informed consent before enrollment. In cases where participants are initially deemed incapable of giving consent due to impairment in decision-making capacity, consent will be reobtained after the participant regains capacity during the study follow-up period (e.g., after recovery from *delirium*). The informed consent form (ICF) contains all requirements elements (Resolution 466, 2012) related to research with human subjects according to the Brazilian National Research Ethics Commission (Conep) and Good Clinical Practice guidelines. The study design is in accordance with the Standard Protocol Items: Recommendations for Interventional Trials (SPIRIT)^(23)^ statement (Table 1S - Supplementary Material), and the trial registry contains all 24 items from the World Health Organization Trial Registration DataSet.

### Study objective

The primary objective is to assess whether the use of dapagliflozin in patients with critical illness and acute organ dysfunction improves the hierarchical endpoint of hospital mortality, initiation of kidney replacement therapy (KRT) and hospital length of stay. The secondary objectives are to assess the effect of dapagliflozin on the individual components of the hierarchical endpoint (hospital mortality, initiation of KRT and hospital length of stay) and on patient-centered endpoints (hospitaland intensive care unit-free days and organ support-free days).

### Study design and population

DEFENDER is an investigator-initiated, multicenter, randomized, open-label, phase 2/3 trial. Eligible participants will be 18 years of age or older, admitted to an ICU with an expected length of stay > 48 hours in the opinion of the attending physician, and with at least one organ dysfunction (hypotension, signs of acute kidney injury, and/or need for new use of a high-flow nasal catheter, noninvasive or invasive ventilation). Patients will be eligible within 24 hours after onset of organ dysfunction. Key exclusion criteria are age < 18 years, pregnancy, end-stage kidney disease on maintenance dialysis, planned ICU admission after elective surgery, use of dapagliflozin or of other SGLT2 inhibitors, total fasting, and type 1 diabetes mellitus or history of diabetic ketoacidosis. A full list of the inclusion and exclusion criteria is presented in table 1. Site investigators and personnel are encouraged to screen all ICU beds on a daily basis to identify potential participants.

**Table 1:** Eligibility criteria

Inclusion criteria	
1. Patients admitted to an intensive care unit with expected length of stay of at least 48 hours in the opinion of the attending physician	
2. Patients with at least one organ dysfunction, defined by at least one of the following:	
- Hypotension (mean arterial blood pressure < 65mmHg or systolic blood pressure < 90mmHg or use of vasopressors - norepinephrine, epinephrine, adrenaline, or vasopressin at any dose)	
- Signs of kidney injury: decreased urinary output within the last 6 hours (< 0.5mL/kg/h for the past six hours) or increase in serum creatinine by at least 0.3mg/dL over previous measurement	
- Need for new use of a high-flow nasal catheter, noninvasive or invasive ventilation	
Exclusion criteria	
1. Pregnancy	
2. Age below 18 years	
3. Patient or legal representative refusal to participate	
4. End-stage kidney disease on maintenance dialysis	
5. Planned intensive care admission after elective surgery	
6. Known allergy to dapagliflozin	
7. Previous use of dapagliflozin or of other SGLT2 inhibitor	
8. Total fasting, unable to receive the medication PO or enterally	
9. Patients with inclusion criterion number 2 for more than 24 hours	
10. Type 1 diabetes or history of diabetic ketoacidosis	

SGLT2 - sodium-glucose cotransporter 2 inhibitor; PO - by mouth.

### Study procedures

#### Interventions

**Figure 1:**
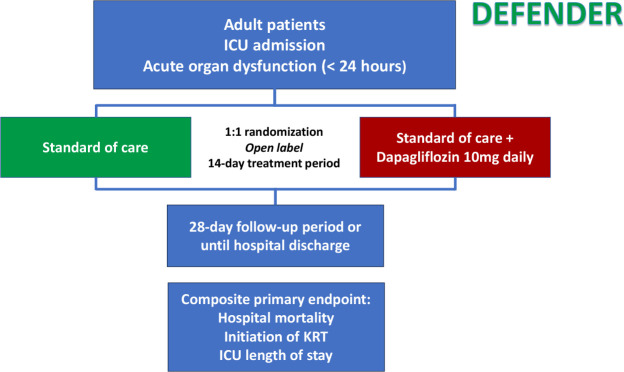
DEFENDER study design and flow chart.

Eligible patients will be randomized 1:1 to dapagliflozin 10mg plus standard of care or standard care alone (Figure 1). Randomization is performed centrally through the Research Electronic Data Capture (REDCap) system,^(24)^ stratified by study site with variable block sizes of 4, 8 and 12. A confidential randomization list was generated by the coordinating center. As an open-label study, no procedures to blind site staff, study personnel, or physicians involved in patient care will be conducted.

ICU - intensive care unit; KRT - kidney replacement therapy

All study procedures will be performed by site staff. Patients in the intervention arm will receive dapagliflozin 10mg/day for 14 days (or until ICU discharge, whichever comes first). Patients in the control arm will receive a routine standard of care. Dapagliflozin will be started on the day of randomization, preferably in the morning without fasting, orally for all participants able to swallow pills. For participants who cannot take oral medications, dapagliflozin will be administered enterally (orogastric tube, oroenteral tube, gastrostomy, jejunostomy, as available) after maceration and dilution in water.

Dapagliflozin should only be withheld in the following situations:

- Requirement for absolute fasting and/or inability to access the enteral route for the drug.
- Occurrence of euglycemic diabetic ketoacidosis, defined by high anion gap metabolic acidosis and ketone bodies in the urine.
- More than one episode of severe hypoglycemia (≤ 50mg/dL) during drug use.
- Withdrawal of consent.
- Suspected allergic reaction to dapagliflozin as well as other idiosyncratic drug reactions, such as DRESS syndrome (drug rash with eosinophilia and systemic symptoms).
- Initiation of kidney replacement therapy.

#### Standard of care

Study sites and investigators will be expected to provide optimal management for critically ill patients according to Brazilian and international consensus and guidelines. This includes but is not limited to ventilation support (oxygen, noninvasive ventilation, mechanical ventilation, among others), hemodynamic support (vasopressors, inotropes), kidney replacement therapy (hemodialysis, hemofiltration, hemodiafiltration, among others), deep venous thromboembolism prophylaxis, delirium, sedation, and pain management. All participant care except for study medication will be solely determined by the local health care team.

#### Monitoring for adverse events

Study sites and investigators will receive specific recommendations on careful monitoring of blood glucose, acid‒base disorders, and renal function during the study to minimize SGLT2 inhibitor-related risks. Glycemic control is to be performed according to the institutional guidelines of each study site for all participants. The study protocol recommends that participants should be monitored at least every 6 hours for blood glucose levels using bedside blood glucose meters or venous/arterial samples until they recover from all organ dysfunctions. For unstable participants who require vasopressors or inotropes or are undergoing invasive mechanical ventilation, blood glucose levels should be monitored at least every 2 hours, but hourly monitoring is strongly recommended, with a target blood glucose level below 180mg/dL. Management of hyperglycemic episodes should preferably be done through an intravenous insulin pump for unstable or mechanically ventilated participants or through intermittent insulin (intravenous or subcutaneous) for other participants. The minimum daily carbohydrate intake for all participants was set at 100g of glucose, considering all infusions. Creatinine levels and blood gas analysis (pH, bicarbonate, anion gap, base excess) will be monitored daily during the first five days of study follow-up.

#### Study follow-up

Participants will be followed for 28 days or until hospital discharge, whichever is sooner. The intervention group will be assessed for adherence to the study drug daily from Days 1 to 14. For both the intervention and control groups, adherence to mandatory laboratory parameters (serum creatinine and blood gas analysis) will be assessed daily from Days 1 to 5.

#### Data collection and management

Trained research personnel from study sites will use the REDCap system to collect data. Data on eligibility criteria that are not met will be collected as screening logs. At randomization, demographic information, comorbidities, concomitant medications, reason for ICU admission, and illness severity will be collected. Daily data collection will include information on treatment adherence, blood gas analysis, and serum creatinine. Blood gas analysis forms will contain ranges of possible values for pH, bicarbonate, and base excess. Information regarding study outcomes will be collected on Day 28 or at hospital discharge. Serious adverse events and adverse events of special interest will be collected throughout the study.

To ensure data quality and confidentiality, investigators and study staff will receive training on data collection, including a dedicated training environment in the electronic data capture system that will register deidentified information about study participants. The coordinating center will check the data weekly, and study sites will receive a monthly report on data quality. On-site and remote monitoring will be conducted during the study. We plan to recruit 500 participants in at least 20 Brazilian ICUs.

### Study outcomes

#### Primary outcome

The primary endpoint is a hierarchical composite of hospital mortality, initiation of KRT, and ICU length of stay up to 28 days after randomization, censored at hospital discharge. ICU length of stay is defined as the total number of calendar days (without fractions) in the ICU from randomization to hospital discharge.

#### Secondary outcomes

Secondary endpoints are hospital mortality, initiation of KRT, ICU-free days, hospital-free days, vasopressor-free days, mechanical ventilation-free days, and KRT-free days up to 28 days after randomization, censored at hospital discharge. To be deemed vasopressorand mechanical ventilation-free, a cutoff of 6 hours or less in an entire calendar day will be used.

Intensive care unit-free days, hospital-free days, vasopressor-free days, mechanical ventilation-free days, and KRT-free days endpoints are defined as the number of calendar days alive and free from each component, measured on an ordinal scale from 0 to 29, with higher values indicating a better outcome. A value of 0 will be assigned to participants who die prior to hospital discharge. Participants who are discharged prior to Day 28 will be assumed to be free of the endpoint through Day 28.

#### Safety outcomes

While meta-analyses of large placebo-controlled randomized trials of SGLT2 inhibitors found the use of this drug class to be generally safe, with a low occurrence of diabetic ketoacidosis (0.3%)^(25)^ and no increased risk for acute kidney injury, hypoglycemia, or hypotension,^(26)^ the DEFENDER study will be the first study involving evaluation of the use of an SGLT2 inhibitor in critically ill patients without COVID-19. Therefore, one of the key aspects of the study is to closely monitor for potential adverse events.

All serious adverse events will be promptly reported by the study sites. Additionally, we will collect information on adverse events of special interest, including liver transaminase elevation (greater than three times above the reference range), skin lesions, hypoglycemia, urinary tract infection, bloodstream infection, and diabetic ketoacidosis, irrespective of their severity and causality assessment. Participants will receive dapagliflozin in a strictly monitored environment, which enables the timely identification and management of potential adverse events. Acid‒base disorders and kidney function will be monitored during the first five days of study follow-up.

### Statistical considerations

#### Planned Statistical Analysis

**Figure 2:**
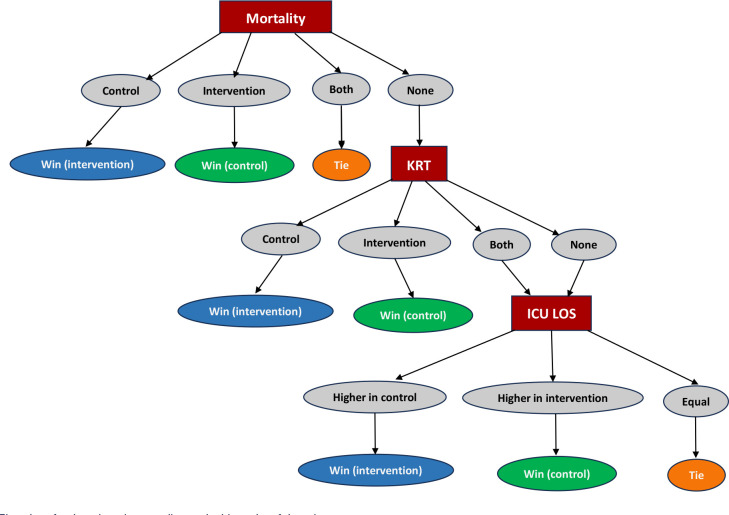
Flowchart for the win ratio according to the hierarchy of the primary outcome.

The primary outcome analysis will be conducted using the unmatched win ratio (WR) method^(27)^ as proposed by Pocock et al.,^(28)^ with the hierarchical composite primary outcome of (1) hospital mortality, (2) initiation of KRT, and (3) ICU length of stay-LOS (in days). The win ratio will be assessed by comparing every possible pair of participants from the intervention and control groups in a pairwise descending fashion. A structural framework using nodes in a decision tree will be used for each level of comparison, with labels of “win” or “loss” assigned if one participant has a better outcome than the other or a “tie” otherwise. Hospital mortality will be the primary level of comparison, and reflecting the higher importance of this outcome, pairwise comparisons in which both participants die will be determined as an early “tie.” If both participants survive, they proceed to the second level of the hierarchy for a comparison of the initiation of KRT. If both participants do not require KRT, or if both require it, the pair is then moved to the third level to compare the ICU LOS. For the ICU LOS outcome, the pair with the shorter duration of stay is considered the “winner.” The structural framework for the three pairwise comparisons according to the hierarchy of the composite primary outcome is shown in figure 2.

The flowchart illustrates how the comparison is made for each potential subject pair, with one participant from the dapagliflozin group and one from the control group. The first node represents comparison of the hospital mortality of the pair, and if both participants die during the study, the result will be deemed an “early tie” and the comparison will end. If both participants survive, they move to the second node for comparison of the initiation of kidney replacement therapy. If both participants do not require kidney replacement therapy, or if both require it, the pair is moved to the third node to compare the intensive care unit length of stay. If the number of days spent in the intensive care unit is equivalent between the two participants, the comparison is classified as a “tie.”

KRT - kidney replacement therapy; ICU - intensive care unit; LOS - length of stay

The WR is calculated as the ratio of the total number of “wins” between the intervention and control groups, and a WR > 1.0 indicates a better outcome in the intervention group. The 95% confidence intervals (95%CI) for the WR will be calculated by bootstrapping based on 10,000 samples, and 95%CI not including the unit (1.0) will be considered statistically significant.

Secondary binary outcomes will be analyzed using a Bayesian hierarchical logistic regression model. The model will include the study group as a predictor and will be adjusted for study site, age, clinical suspicion of sepsis, use of vasopressors, and mechanical ventilation at randomization. A neutral prior of moderate strength^(29)^ and normal distribution, centered at an odds ratio (OR) of 1.0 and a standard deviation of 0.35, corresponding to a 95% probability that the effect (OR) is within the 0.5 - 2.0 range, will be used. Superiority of the dapagliflozin group over the control group will be determined if the posterior distribution of the adjusted odds ratio (aOR) being less than 1.0 [Pr (aOR < 1.0)] is more than 95%. The results will be presented as the posterior distribution of the aOR (in log scale), a 95% credible interval, and the probability of the aOR being less than 1.0.

Intensive care unit-free days, hospital-free days, vasopressor-free days, mechanical ventilation-free days, and KRT-free days will also be analyzed using a Bayesian hierarchical ordinal model, using the same covariates from the binary outcomes model. Frequentist analysis exploratory analysis for secondary outcomes will also be performed, and no p values will be presented. Binary outcomes will be analyzed thorough a logistic regression model, with the same covariates as the Bayesian models, and data will be presented as aOR, crude OR, and corresponding 95%CI. Treatment differences in free day outcomes will be calculated using the Hodges‒Lehmann method and presented as the difference in days and 95%CI.

All primary, secondary and safety analyses will follow the intention-to-treat principle. A sensitivity safety analysis will also be conducted in all participants who received at least one dose of dapagliflozin (safety population). Full details of all planned analyses are provided in version 1.0 of the Statistical Analysis Plan (Supplementary Material). All analyses will be performed using R software (R Project for Statistical Computing).

#### Subgroup analysis

For the primary and secondary outcomes, the following relevant subgroup analyses were planned: clinical suspicion of sepsis at randomization (yes/no), diabetes mellitus (yes/no), serum creatinine at enrollment (< 1.5mg/dL, 1.5 - 3.0mg/dL, and > 3.0mg/dL), cardiovascular reason for ICU admission (yes/no), and age (< 65 and ≥ 65 years). Stratified WR analysis will be performed for each subgroup stratum. The 95%CIs will be calculated by bootstrapping 10,000 samples for each analysis, and 95%CIs not including the unit (WR = 1.0) will be considered statistically significant. We will also perform additional analysis for the secondary outcomes within each subgroup using the same Bayesian models and exploratory frequentist analysis.

#### Power and sample size

**Figure 3:**
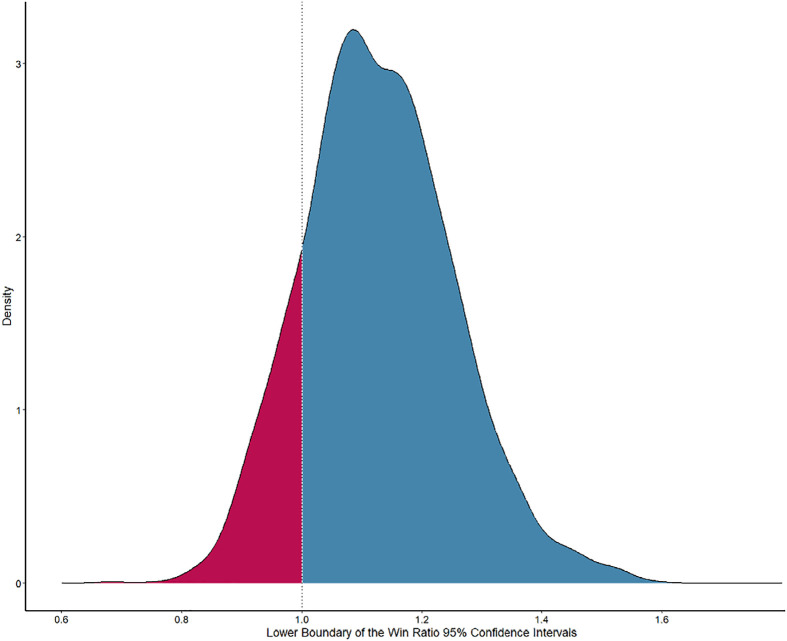
Lower boundary of the win ratios 95% confidence intervals obtained through 10,000 simulations on samples of 500 patients.

We estimated that a sample size of 500 participants would provide at least 85% power to detect a win ratio and corresponding 95%CI above 1. This calculation was made based on the following assumptions: (1) a 2% absolute reduction in hospital mortality from 30 to 28% with dapagliflozin; (2) a 3% absolute reduction in initiation of KRT from 10 to 7% with dapagliflozin; and (3) a mean reduction in ICU LOS of 0.5 days with dapagliflozin. The estimation was obtained after performing 10,000 simulations in samples of 500 participants using these assumptions and 95% confidence intervals obtained by bootstrapping. Figure 3 displays the results of the obtained lower boundaries of the 95% confidence intervals of the win ratio values from these simulations.

The density plot displays the distribution of the lower boundaries of win ratio values from 10,000 simulations, with the confidence interval obtained after 1,000 bootstraps from the simulated data. The dashed line indicates win ratios with 95% confidence intervals above 1.0. The blue area represents simulations where the win ratio’s 95% confidence interval was greater than 1.0, while the red area represents simulations where the 95% confidence interval was less than 1.0.

### Interim analysis and Data and Safety Monitoring Board

The DSMB regularly reviews unblinded patient-level data during the study to ensure participant safety. The first safety analysis occurred on March 27, 2023, after 100 participants were enrolled. An interim analysis will occur when half of the sample size (n = 250) has been enrolled and followed for at least 14 days. For this specific analysis, trial recruitment will be halted for four weeks and will resume only after the DSMB deliberation. Another safety analysis is planned to occur after 375 participants are enrolled. The sole purpose of the interim analysis is to assess safety, and the trial will not be halted for benefit or futility. The frequency of safety interim analysis might be changed if deemed appropriate by the DSMB members.

At the interim analysis, the DSMB will use a Bayesian framework (simple logistic regression) to assess the posterior probability distribution for harm (OR > 1.0) for hospital mortality and initiation of KRT. If this probability is greater than 80%, the DSMB will recommend stopping the trial. Harm will also be assessed in two key subgroups: participants with hypotension or acute kidney injury at randomization. If the probability of harm is greater than 80% for either subgroup, the DSMB will recommend excluding them from the trial.

Additional interim analyses may be convened at the discretion of any DSMB member if new scientific data or concerns arise during the study, especially regarding the ongoing SGLT2i arms of the ACTIV-4A (NCT04505774) and RECOVERY (NCT04381936) platform trials. Based on the interpretation and quality of the data, the number of events, and other factors, the DSMB may recommend modifications to the protocol (e.g., changes to inclusion/exclusion criteria), suspension, or termination of the trial, and advise the Steering Committee. However, all final decisions regarding trial conduct will be at the discretion of the Steering Committee.

### Current status

The first participant was randomized in November/2022, and there are currently 14 active and 13 enrolling sites. The list of active and enrolling sites is updated on a monthly basis and can be accessed publicly on the study clinicaltrials.gov webpage.

## DISCUSSION

DEFENDER will be the first study to test the hypothesis that SGLT2 inhibitors can reduce organ dysfunction and mortality of critical illness from different etiologies. This approach is different from that of several other randomized clinical trials that failed to demonstrate improved outcomes in ICU patients, such as the use of vitamin C^(30-32)^ and statins for sepsis,^(33)^ probiotics for ventilator-associated pneumonia,^(34)^ among others.^(35)^

Sodium-glucose cotransporter-2 inhibitors have been used in more than 45,000 participants in over 13 large-scale trials, and the evidence overwhelmingly suggests that the cardiovascular and kidney benefits outweigh the low risk of serious harm.^(25)^ It is biologically plausible that the effects of drugs in this class may positively impact pathways of organ dysfunction during acute critical illness.^(36)^ Furthermore, the initiation of SGLT2 inhibitors in hospitalized patients with acute heart failure during placebo-controlled randomized clinical trials was safe and did not increase the risk of hypotension, hypoglycemia, or acute kidney injury.^(26)^

Given that this is the first time that SGLT2 inhibitors will be used in general ICU patients, we have implemented a series of safety measures to minimize potential risks and preventable harm for current and future study participants that will permit a comprehensive understanding of the risk/benefit profile of dapagliflozin in critically ill patients. In conclusion, DEFENDER presents a new approach to evaluate the potential of SGLT2 inhibitors in reducing organ dysfunction and mortality in critically ill patients and will provide valuable information for future trials in this population.
